# More pests but less pesticide applications: Ambivalent effect of landscape complexity on conservation biological control

**DOI:** 10.1371/journal.pcbi.1009559

**Published:** 2021-11-08

**Authors:** Patrizia Zamberletti, Khadija Sabir, Thomas Opitz, Olivier Bonnefon, Edith Gabriel, Julien Papaïx

**Affiliations:** 1 INRAE Biostatistique et Processus Spatiaux, INRA-PACA, Avignon, France; 2 Institute of Horticultural Production Systems, Leibniz University Hannover, Hannover, Germany; University of Wisconsin, UNITED STATES

## Abstract

In agricultural landscapes, the amount and organization of crops and semi-natural habitats (SNH) have the potential to promote a bundle of ecosystem services due to their influence on ecological community at multiple spatio-temporal scales. SNH are relatively undisturbed and are often source of complementary resources and refuges, therefore supporting more diverse and abundant natural pest enemies. However, the nexus of SNH proportion and organization with pest suppression is not trivial. It is thus crucial to understand how the behavior of pest and natural enemy species, the underlying landscape structure, and their interaction, may influence conservation biological control (CBC). Here, we develop a generative stochastic landscape model to simulate realistic agricultural landscape compositions and configurations of fields and linear elements. Generated landscapes are used as spatial support over which we simulate a spatially explicit predator-prey dynamic model. We find that increased SNH presence boosts predator populations by sustaining high predator density that regulates and keeps pest density below the pesticide application threshold. However, predator presence over all the landscape helps to stabilize the pest population by keeping it under this threshold, which tends to increase pest density at the landscape scale. In addition, the joint effect of SNH presence and predator dispersal ability among hedge and field interface results in a stronger pest regulation, which also limits pest growth. Considering properties of both fields and linear elements, such as local structure and geometric features, provides deeper insights for pest regulation; for example, hedge presence at crop field boundaries clearly strengthens CBC. Our results highlight that the integration of species behaviors and traits with landscape structure at multiple scales is necessary to provide useful insights for CBC.

## Author summary

In the agricultural context, the loss of semi-natural surfaces often results in high pest abundance requiring elevated pesticide loads. Habitat heterogeneity resulting from the agricultural intermixing of arable fields and semi-natural areas is key to allow organism fluxes across agro-ecological interfaces by influencing ecological processes. Semi-natural habitats (SNH) are often restricted to linear structures, such as hedgerows, but they play an important role by hosting a large number of species. However, the effect of hedgerows is controversial, as it could result in a positive, ineffective or negative effect for CBC. Usually, the impacts of landscape structure on pest population dynamics and resulting CBC are assessed through field experiments with a specific focus, which cannot be generalized, lack flexibility and are limited by the need to manipulate relatively large landscapes. Here, we tackle the challenge to investigate the controversial role of semi-natural habitats for CBC by presenting a simulation-based approach, which allows us to characterize the joint influence of landscape structure and species traits on CBC service. Our study corroborates that spatial heterogeneity, species traits and their interactions are fundamental for CBC. We show that hedge presence alone is not sufficient to lead to strong pest reduction, but hedge-based predators help to maintain the pest density under the pesticide threshold. Instead, SNH presence coupled with appropriate predator traits leads to stronger decrease of pest population. Moreover, we highlight an important scaling effect of SNH, which at the local scale has an even more important impact on CBC as local properties are considered.

## 1. Introduction

Agricultural landscape simplification results in substantial loss of semi-natural mosaics and of non-crop field margins. It is often associated with high pest abundance, which in turn requires a higher pesticide input [1,2]. Consequently, a negative relationship emerges between intensity of agriculture and agricultural landscape biodiversity [3] because of a partial replacement and suppression of the ecological services provided by communities of beneficial organisms [4,5]. Habitat heterogeneity is key to allow cross-system fluxes of organisms across agro-ecological interfaces by influencing ecological dynamics within those habitats [6,7] and potentially increasing predator abundance and diversity in agricultural systems [8,9]. In addition, complex landscape favours habitat and resource diversity for predators thanks to increased availability of alternative preys, higher microclimate heterogeneity, the presence of refuges from their own predators and for overwintering [10]. In arable land, semi-natural habitat (SNH) is typically restricted to hedgerows. These linear structures play an important role as relatively perennial line corridors because of their temporal stability with respect to crop fields. Their presence supports predator dispersal and movement to escape from disturbances and to find food resources scattered in time and space [11,12].

While SNH favours the presence or abundance of functional groups of organisms in landscapes, it can also result in ineffective conservation biological control (CBC) [12,13] with no, or even negative effects on pest control [12–14]. A meta-analysis revealed that pest pressure in complex landscapes is reduced in 45% of cases, not affected in 40% of cases and increased in 15% of cases [9]. The analysis in [15] highlights the difficulty of stating general and systematic pest and predator interactions and responses; it is based on a very large pest control dataset from which a remarkable variability in pest and enemy responses to different landscape metrics is found. For example, the effect of landscape structure on pests remains inconclusive, as many crop pests also benefit from nearby non-crop habitat [12–14]. It may occur that SNH offers more complementary resources to pests rather than to predators to complete their life cycle [6]. Predator abundance is not always enough to guarantee a consistent reduction of pest species [16] in case of the presence of alternative prey (known as *dilution effect*) [17], or increased intra-guild predation [18]. Life history traits, in particular those traits related to mating systems, competitive skills, movement abilities and habitat use, are also of major importance by affecting species’ responses to landscape heterogeneity and being readily linked with ecological processes [19]. Thus, effect direction and magnitude jointly depend on organisms and landscapes under study [20,21].

In general, the impacts of landscape structure on pest population dynamics are investigated through empirical correlative approaches with global descriptors at landscape level, due to the difficulty of manipulating large landscapes for local analyses and due to the lack of the spatio-temporal dimension. The main drawback of these approaches is the difficulty of linking correlation levels to population dynamic processes, such as local population growth or migration behavior [22]. A complementary approach, combining theoretical modeling and computer simulations, consists in coupling generative landscape models with population dynamics models to explore how different landscape configurations, including the hedge network structure, affect CBC [23].

A major goal of this work is to implement a general simulation-based approach to obtain theoretical insights on CBC by incorporating landscape effects and species traits, which can serve as basis to formulate practical recommendations. In order to assess what are the main factors that influence the predator-pest population densities in complex landscapes, following questions are investigated: (i) Can landscape composition and configuration reduce the number of pesticide applications by enhancing CBC? (ii) How do species traits related to dispersal, predation and population demography modify the effect of landscape heterogeneity? Specifically, we develop a stochastic landscape model to simulate realistic agricultural landscape compositions and configurations of fields and linear elements for crop and semi-natural allocation. The generated landscapes are used as spatial support over which we simulate spatially explicit predator-pest dynamics. The population model accurately links 2D diffusion on surface, 1D diffusion on linear elements, and the flux interchanges among them to put particular attention on the linear element integration; see S1 and S2 Videos. Predators use hedges as their natural habitat where their population naturally develops, but they can also move into crop field to feed on pests. Pests consider crop fields as their natural habitats where they show positive growth, while they are not influenced by hedge elements. Our study explores how the joint consideration of spatial heterogeneity, landscape structure, species traits and their interactions helps to achieve effective CBC. We present and discuss results in the following sections; the technical description of our model and statistical methods is given in Model and method section.

## 2 Results

### 2.1 Sensitivity of predator density, pest density and pesticide applications to model parameters

**Fig 1:**
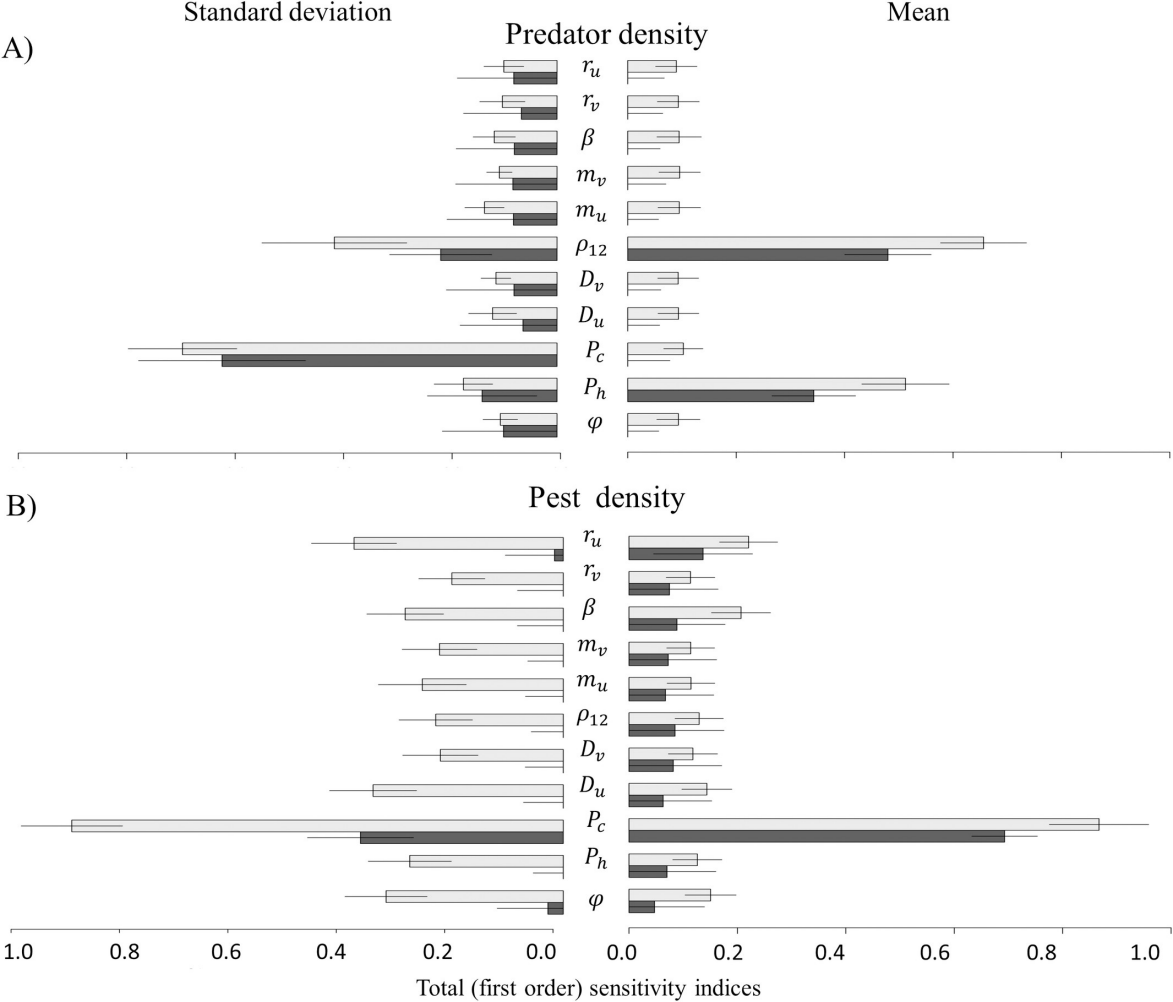
Sobol sensitivity analysis. Total sensitivity indices (light grey bar) and first-order sensitivity indices (black bar) of space-time averaged values for predator density (a) and pest density (b), based on the mean (right) or on the standard deviation (left) calculated over replicated simulations. The length of the bar indicates the mean of the sensitivity index, and the solid line indicates its 95% confidence interval.

Fig 1 shows the results of a Sobol sensitivity analysis, where sensitivity indices are denoted by *I*_*variable*_ in the following and are calculated from replicated simulations with the same underlying parameter configuration. The sensitivity analysis of the mean of model outputs across landscape replicates (Fig 1A right) shows that variations in mean predator population density are mainly explained by predator migration ($I_{\rho_{12}}=50\%$) and by the proportion of hedges ($I_{P_{h}}=41\%$), whereas interactions among parameters have little impact on the outputs. For the mean pest population density and the average number of pesticide applications, crop proportion ($I_{P_{c}}=78\%$ and $I_{P_{c}}=83\%$, respectively) and pest growth rate ($I_{r_{u}}=17\%$ and $I_{r_{u}}=15\%$, respectively) are the most important parameters to explain model output variability, again with only little interaction between model parameters (Fig 1B right). Complete results for pesticide applications are given in the S1 Text.

The sensitivity analysis of standard deviation of model outputs across landscape replicates gives different importance to the input variables as compared to the mean values. For the predator density, crop proportion (*P*_*c*_), predator migration (*ρ*_12_), hedge proportion (*P*_*h*_) and spatial crops and hedges aggregation (*φ*) explain respectively 55%, 19%, 9% and 9% of the variability of model outputs (Fig 1A left). For the pest and pesticide applications, results are consistent with the results obtained for the mean. However, interactions between model parameters are important to explain variations in the standard deviation of predator and pest density, as well as of pesticide applications among landscape replicates. This implies that particular landscape structures, characterized by a combination of several descriptors, have to be considered to fully understand the drivers of predator-pest dynamics.

### 2.2 Landscape structure effects on the predator-pest dynamics

Estimated coefficients of landscape variables (denoted *E*_*variable*_ in the following) on predator density highlight a positive effect of hedge proportion ($E_{P_{h}}=0.40\pm 0.05$), a negative effect of crop proportion ($E_{P_{c}}=-0.20\pm 0.04$) and a positive interaction among both variables ($E_{P_{h}:P_{c}}=0.08\pm 0.02$), which implies that hedges can buffer the negative effect of increased crop proportion. Migration from hedges to fields ($E_{\rho_{12}}=0.56\pm 0.01$) has the highest positive effect on predator density with again a positive interaction with crop proportion.

As expected, crop proportion $(E_{P_{c}}=1.50\pm 0.16),$ as well as spatial crop and hedge aggregation (*E*_*φ*_ = 0.55 ± 0.02), have a strong positive effect on pest density. Both variables interact negatively ($E_{\varphi :P_{c}}=-0.11\pm 0.01$), as high aggregation results in an increase of the size of contiguous crop fields, which lowers the effect of increased crop proportion. The positive effect of crop proportion is lowered by its interaction with hedge proportion $\left(E_{P_{h}:P_{c}}=0.03\pm 0.06\right)$ and also with predator migration from hedge to fields ${(E}_{P_{c}:\rho_{12}}=0.06\pm 0.06)$. Counterintuitively at first sight, an increase in hedge proportion $\left(E_{P_{h}}=0.09\pm 0.11\right)$ has a positive effect on pest density. Indeed, predator presence over all the landscape helps to stabilize the pest population by keeping it under the thresholds that would trigger a pesticide application. This is further confirmed by the fact that hedge proportion ($E_{P_{h}}=0.32\pm 0.57$), predator spillover from hedges to fields ($E_{\rho_{12}}=0.61\pm 0.34$) and concurrence of high crop proportion and aggregation ($E_{\varphi :P_{c}}=0.24\pm 0.09$) have a positive effect on the presence of pesticide applications, but a negative effect on pesticide application numbers ($E_{P_{h}}=-0.11\pm 0.07,\;E_{\rho_{12}}=-0.19\pm 0.08,\;E_{\varphi :P_{c}}=-0.07\pm 0.01$).

Among species traits, predator migration from hedges to fields ($E_{\rho_{12}}=-0.13\pm 0.12$) has the highest negative impact on pest density. Pest diffusion ($E_{D_{u}}=-1.03\pm 0.01$), due to a dilution effect, and the predating rate (*E*_*β*_ = −0.24 ± 0.01), have also a negative impact on the pest, while the growth rate ($E_{r_{u}}=0.41\pm 0.01$) contributes positively to pest density. Fig F in S1 Text shows all estimated effects and their confidence intervals for predator and pest density and pesticide application presence/absence and number, see also Table 1.

**Table 1:** Estimated coefficients (only those discussed in the text). Estimated coefficient on predator and pest density (left) and on the presence/absence (P/A) and number (No.) of pesticide applications (right) at landscape and patch level. + indicates a positive effect, – a negative effect, NS a non significant effect.

		Density				Pesticide application	
	Coefficient	Predator	Pest		Coefficient	P/A	No.
***Landscape***	$E_{P_{h}}$	+	+	***Landscape***	$E_{P_{h}}$	+	-
	$E_{P_{c}}$	-	+		$E_{\rho_{12}}$	+	-
	$E_{P_{h}:P_{c}}$	+	+		$E_{\varphi :P_{c}}$	+	-
	$E_{\rho_{12}}$	+	-	***Patch***	*E*_*area*_	-	-
	$E_{\varphi :P_{c}}$	NS	-		*E*_*perim*_	-	+
	$E_{P_{c}:\rho_{12}}$	+	+		$E_{{Adj}_{C}}$	+	+
	$E_{D_{u}}$	+	-		$E_{{Adj}_{H}}$	-	-
	*E*_*β*_	+	-		$E_{{Adj}_{Tr}}$	+	+
	$E_{r_{u}}$	+	+				

By checking the sensitivity of our results with respect to the pesticide application variables (*i*.*e*., pesticide application efficacy [optimal vs realistic] and pesticide thresholds [low vs high], see S1 Text), we find that there is no variation of the direction of the estimated effects, but the magnitude of the effect can increase or decrease depending on the scenario considered. Specifically, when pest reduction is lower due to low pesticide efficacy, or, when pest reduction is slower due to an elevated pesticide threshold, hedges show a more important effect in slowing down pest dynamics thanks to predator presence providing a more efficient CBC.

### 2.3 Effect on pesticide application at local scale

Locally, presence of pesticide applications is negatively influenced by field area and perimeter (*E*_*Area*_ = −0.32 ± 0.01, *E*_*Perimeter*_ = −0.10±0.03). These effects reflect both a slower pest diffusion in large fields and higher predator incoming fluxes to fields with long perimeter. Conversely, when pesticide applications occurred in a field, the total number of pesticide applications increases with field perimeter due to spillover form the neighborhoods. An increase in the number of adjacent crop fields produces a positive effect on the presence ($E_{{Adj}_{C}}=0.74\pm 0.01$) and number ($E_{{Adj}_{C}}=0.20\pm 0.002$) of pesticide applications, while an increase in the number of adjacent hedges leads to a negative effect on the presence ($E_{{Adj}_{H}}=-0.07\pm 0.01$) and number ($E_{{Adj}_{H}}=-0.05\pm 0.001$) of pesticide applications. Whereas in the global model the increase of hedge proportion is associated with a positive effect on the presence of pesticide applications, we attribute the negative effect at local level to the fact that the predator tends to locally maintain the pest density under the pesticide threshold, especially after a first pesticide application. The number of pesticide applications in adjacent fields is positively correlated to their local presence ($E_{{Adj}_{Tr}}=2.99\pm 0.01$) and number ($E_{{Adj}_{Tr}}=0.13\pm 0.001$), indicating local proliferation of the pest. Fig G in S1 Text shows all estimated local effects and confidence intervals for pesticide application presence/absence and number, see also Table 1.

## 3 Discussion

Sustainable management of pests and diseases in agro-ecosystems requires a better understanding of how landscape structure drives and alters population dynamics. By simulating different landscape configurations including linear corridors, and the predator-pest dynamics, the present research aims at characterizing the joint influence of landscape structure and species traits on CBC service. Our study corroborates that spatial heterogeneity, landscape structure (i.e., the size and physical arrangement of patches), species traits and their interactions play a key role for CBC.

High crop proportion is the major determinant of increasing pest population and results in an increased number of pesticide applications over the whole landscape. Indeed, increasing crop proportion in fragmented landscapes ensures food availability to the pest all over the landscape [1,2,12]. In highly aggregated landscapes, the size of contiguous crop patches is already large enough to sustain a relatively large pest population, thus lowering the effect of an increase in crop proportion [14]. The effects of crop proportion and spatial crop and hedge aggregation are intimately linked to pest growth rate and dispersal capability. Indeed, unfavorable landscape properties for the pest (i.e., low proportion and high fragmentation) can be compensated by a higher growth rate. However, the effect of dispersal is a double-edged sword since high dispersal helps spreading on fragmented landscapes but comes with a larger amount of propagules lost in unsuitable habitats, potentially leading to a dilution effect [3,24,25].

As expected, hedge proportion (i.e., SNHs) positively affects predator presence in agricultural landscapes. In addition, the predator’s ability to move between SNHs and crop habitats is the parameter that increases most strongly the predator density, since it enables predators to reach complementary resources in crop fields more easily. Predator fluxes from adjacent habitat is reported to have a major impact on pest populations in crop fields [3,12,26]. Spillover from hedges to fields not only depends on predator propensity to forage outside their natural habitat, but also on semi-natural patch connectivity and on crops and predator reservoir interface [27]. Thus, different combinations of SNH proportion and aggregation influence landscape structural connectivity and are also important determinants of predator efficiency in regulating crop pests [27].

In our representation, hedges are modeled as a source of predators where these have logistic growth. This is a simplification for predator dynamics in their natural habitat, as we do not consider potential prey presence in hedges and predator foraging behavior in crop fields. For example, the growth rate, instead of being constant, could depend on the time spent in the fields and on the number of consumed preys. In addition, predating rate and consumption rate are crucial in determining the efficiency of CBC [28]. Here, these parameters are not identified as influential in the dynamics, maybe because they are assumed identical (parameter *β* in our model). Finally, another strong assumption of our model is that we refer to a selective pesticide application which does not affect predator mortality, such that we do not explore a broad-spectrum pesticide scenario. In general, broad-spectrum pesticides are more commonly applied [17], but there are pest management programs where selective insecticides have been proved to be particularly effective in combination with a CBC strategy by weaving together direct targeted reduction in pest numbers with predator conservation [17,29]. Moreover, introducing broad-spectrum pesticide application effects may result in secondary pest breakouts [30–32], where pests benefit from the predator reduction. Then, additional pesticide loads would be necessary to decrease pest density, which in turn continuously decimates the predator population [33]. Therefore, the effect of SNH and predators, and their relationships for CBC outcomes, would be confused and masked. In our work, an indirect effect could be observed: in crop fields, a positive predator growth rate relies only on pest availability, such that a strong pest reduction due to pesticide applications is automatically translated into a strong impact on predator density when such pesticide applications occur.

In our analysis, we found that the predator’s ability to disperse from hedges to crop fields has a major influence on pest density and related pesticide applications. High crop proportion enhances pest density, but this effect is counter-balanced by the joint effect of hedge proportion and predator spillover from hedges to fields, which favors predator pressure and reduces pesticide applications. Indeed, hedges ensure an increased functional landscape connectivity, which enables predators to successfully disperse and feed on complementary resources in the fields. Interestingly, however, we found that if SNHs can sustain a high population of predators [25], this is not sufficient to achieve a decrease in pest density. Indeed, by keeping the pest population density under the pesticide application threshold, the predator population can favor its spread across the landscape, thus increasing pest density at the landscape scale, even if fewer pesticide applications are applied. Most of the studies consider the amount of SNH as a proxy for predator presence and focus on how landscape structure directly influences CBC. However, as highlighted by our results (see also [34]), the extent to which species are influenced by landscape heterogeneity depends on their traits. For example, [35] argue that predators with an oriented movement are better able to deliver pest control services. They discuss the interplay among predator mobility, proportion of crop and SNHs. More generally, predator fluxes from SNH are expected to be particularly strong when (i) predator attack rates on prey are high, (ii) predator movement abilities are substantial, and (iii) predator mortality rates in the recipient habitat are low [34]. However, we point out that the predator we model is a generalist predator that does not show strong aggregation behaviour towards pests. Pests represent a predator resource in field, but predators can persist in the landscape also without pests as they have a positive growth in hedges. Different outcomes would be probably observed when considering a specialist predator showing an aggregating behaviour around local pest outbreaks [36]. As for example in [36], specialist predators are found to be more effective agents in suppressing local outbreaks than generalist ones.

The amount of predator spillover from hedges to fields, and the distance over which pest and predator can spread, both depend on local configurational variables such as field size, shape, amount of shared edge, and connectivity [20]. Large fields can support high pest volumes, but it has been demonstrated that the relationship between field size and pest density can take several forms depending on assumptions, conditions and species considered [37]. Our results show a negative effect of large field area on the need and quantity of pesticide applications, which, according to [37], may come from the elevated growth rate of the prey combined with its good dispersal ability. By contrast, a high number of pesticide applications is favoured by long field perimeters, as these facilitate high fluxes of pest coming in from surrounding fields. However, when a hedge is present on a field boundary, we observe a reduction in numbers of pesticide applications, as there is an increase of predator spillover from hedges into fields [9]. Interestingly, we show a contrasted effect of hedges depending on the scale considered. At global scale, the proportion of hedges shows a positive effect on pest density and has a negative effect only on the presence of pesticide application. At local scale, an elevated number of hedges on crop boundaries shows an even more important impact on CBC by negatively affecting both the local presence and number of pesticide applications [25].

Landscape simplification is a major driver of pest abundance and consequently has strong impacts on the necessity of pesticide applications and their frequency. We find that natural habitat enhances predator population, but it does not systematically translate into a strong correlation with pest density decrease. However, a relatively high predator density often helps maintaining pest density below the threshold level above which pesticides are applied, thus preventing highly localized pest densities. However pest population can already have a moderate density level over substantial surfaces and therefore may quickly propagate in every point of the space. Indeed, in our model the hedges are generally expected to play a positive role, but our results at global scale show that the final outcome must be analyzed in a much more nuanced way. By contrast, predator spillover from hedges to fields is fundamental for CBC; it reduces pest density and guarantees high predator fluxes and different habitat connectivity. At field scale, landscape geometric features, hedge presence and habitat connectivity are able to influence predator-pest dynamics, and therefore they affect the number of pesticide applications. This highlights the importance of conducting a multi-scale analysis to consider the differences in outcomes at landscape and patch scale for pest CBC [14]. In most of our analyses, we considered global outputs by averaging pest and predator densities over crop fields. However, populations are obviously structured in space and time. Thus, a complementary analysis studying how landscape structure impacts spatio-temporal predator-pest dynamics would bring deeper insights on pest outbreak determinants. Moreover, a larger number of pest and predator species, inter/intra-species interactions and also different trophic network structures, could be considered in future work to better understand the role of pest and predator diversity on CBC efficacy.

## 4 Models and methods

### 4.1 Stochastic landscape model

The landscape is represented through a vectorial approach, which is appropriate for representing the highly regular geometric patterns of agricultural landscapes [38,39]. It is composed of polygons representing fields, separated by edges. Landscape elements are characterized by their geometry (e.g., vertex coordinates, size and shape), and by categorical information defining the land-cover (e.g., crop or natural habitat). The landscape geometric structure is fixed and based on a real landscape with an extent of 5.55 km. The landscape is transformed into a T-tessellation [40,41] composed of 188 polygons with a total of 577 edges.

**Fig 2:**
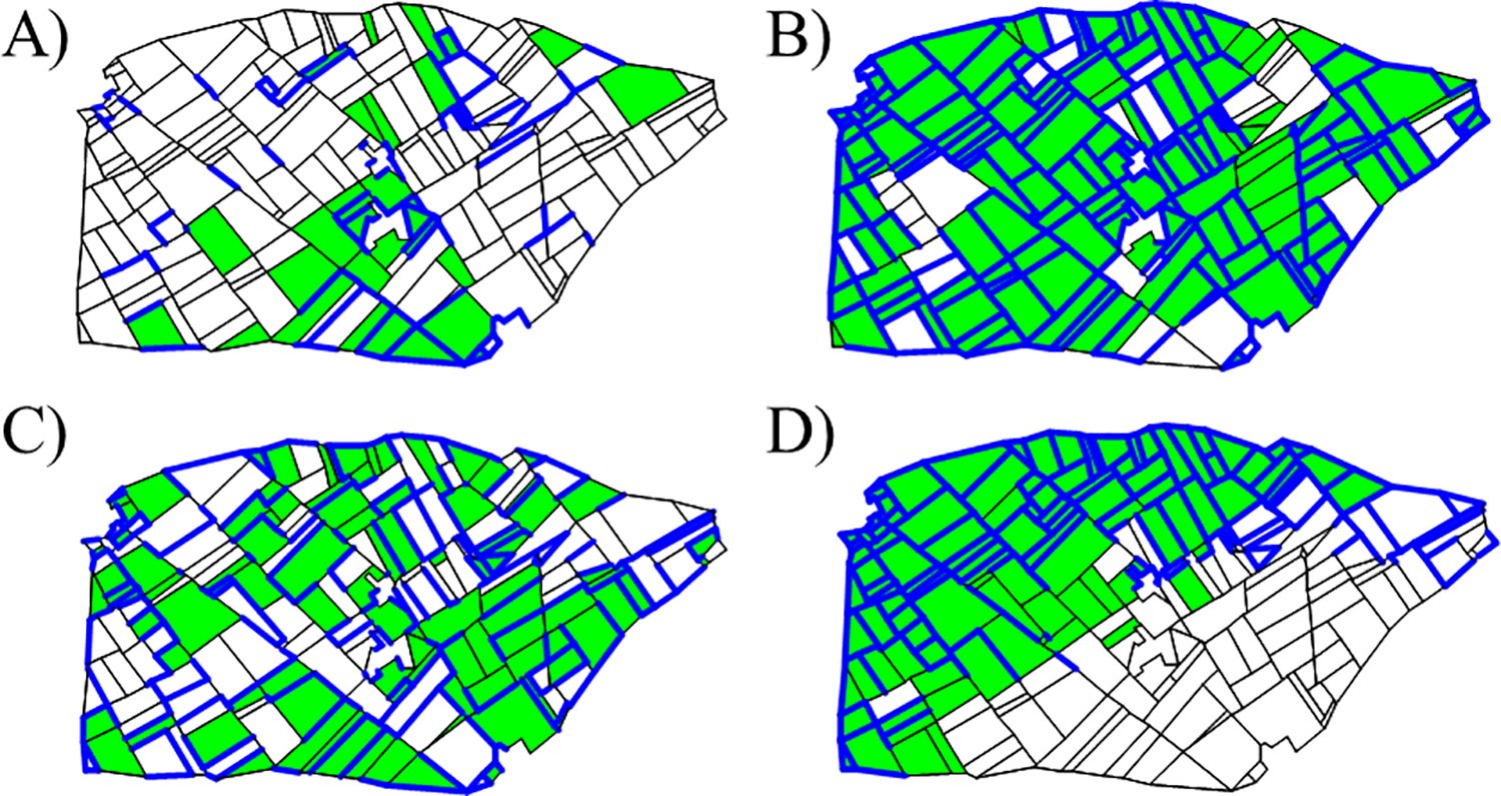
Simulation examples. Examples of simulated landscape structures with interacting elements using the following allocation categories: for fields, (i) crop (green) and (ii) non-crop (white); for edges, (i) hedge (blue) and (ii) no-hedge (black). First row: low (a) and high (b) proportions of crop and hedges (0.2 and 0.8, respectively), with fixed parameter configuration for aggregation and fixed correlation between crop and hedges (0.5). Second row: low (c) and high (d) crop and hedge aggregation level from left to right, with fixed proportion of crop and hedges (0.5) and fixed correlation between crop and hedges (0.5).

We use Gaussian random fields (GRFs) to allocate a proportion of polygons and edges as crops representing the principal culture and hedges to provide SNHs, respectively. A threshold on the simulated GRF values is set to attribute specific landscape elements depending on the value being below or above the threshold. By definition, a GRF denoted by *W* is a random surface over continuous 2D space, for which the multivariate distribution of the values (*W*(*x*_1_), *W*(*x*_2_),…,*W*(*x*_*n*_)) observed at a finite number of locations *x*_1_, *x*_2_,…,*x*_*n*_ in the landscape corresponds to a multivariate normal distribution, characterized by its mean vector and its covariance matrix Σ. The mean is fixed to 0 and the exponential correlation function is used for Σ, such as $\Sigma_{ij}=e^{-(\frac{\left|x_{i}-x_{j}\right|}{\varphi })}$, where |*x*_*j*_−*x*_*i*_| is the Euclidean distance between any two points *x*_*j*_ and *x*_*i*_. The range parameter *φ*≥0 governs the strength of clustering of category allocation to landscape elements. To handle the interactions between the allocation of hedge and crop, we simulated two correlated GRFs for crop (*W*_*c*_(*s*)) and hedge (*W*_*h*_(*s*)):

$$W_{c}(s)=\rho W_{h}(s)+\sqrt{1-\rho^{2}}\tilde{W}(s),$$ (1)

where *ρ*∈[−1, 1] controls the correlation between *W*_*h*_ and $\tilde{W}$, which is a GRF independent from *W*_*h*_. The parameter used for the landscape models with their range of values can be found in Table 2. Fig 2 shows an example of four landscapes simulated according to different proportions and aggregation levels of hedges and crop fields.

**Table 2:** Description of parameter values. *Spatial aggregation is the parameter controlling the adjacency of crop elements among each other and hedge elements among each other.

Parameters	Description	Values		Units	References
		Min	max		
**For landscape model**					
*φ*	Spatial aggregation of hedges and crops*	5.55/100	5.55	km	
P_c_	Proportion of crop	0	1	-	
P_h_	Proportion of hedges	0	1	-	
*ϱ*	Correlation between crops and hedges *GRF*_*S*_	0.5		-	
**Parameters for population dynamic mode**					
$D_{2}^{v}$	2D diffusion rate of the predator	0.000625	0.012	*km* ^2^ *d* ^−1^	Corbett et al., 1996; Pearce et al. 2006
1/*m*_*v*_	Lifespan of the predator	20	66	*d* ^1^	Pearce et al. 2006
*β*	Predating rate	0.01	0.010	*pest* ^−1^ *d* ^−1^	Pearce et al. 2006
*ρ*_21_	Migration rate of the predator from field to hedge	0.05		*km* ^−1^ *d* ^−1^	
$D_{1}^{v}$	1D diffusion rate of the predator	0.012		*km* ^2^ *d* ^−1^	Corbett et al., 1996; Pearce et al. 2006
*r*_*v*_	Intrinsic growth rate of the predator on hedges	0.010	0.020	*d* ^−1^	Xia et al., 1999
$K_{h_{i}}$	Carrying capacity of hedges for the predator	1		*predators km* ^−2^	
*ρ*_12_	Migration rate of the predator from hedge to field	0	0.05	*d* ^−1^	
*D* ^*u*^	2D diffusion rate of the pest	0.000625	0.012	*km* ^2^ *d* ^−1^	Corbett et al., 1996; Pearce et al. 2006
*r*_*u*_	Intrinsic growth rate of the pest	0.010	0.020	*d* ^−1^	Xia et al., 1999
*C*_*it*_	Carrying capacity of 2D system for the pest	20 (without pesticide) 0.1 (after pesticide)		*pests km* ^−2^	
1/*m*_*u*_	Lifespan of the pest	20	66	*d*	Pearce et al. 2006

### 4.2 Predator-pest model

**Fig 3:**
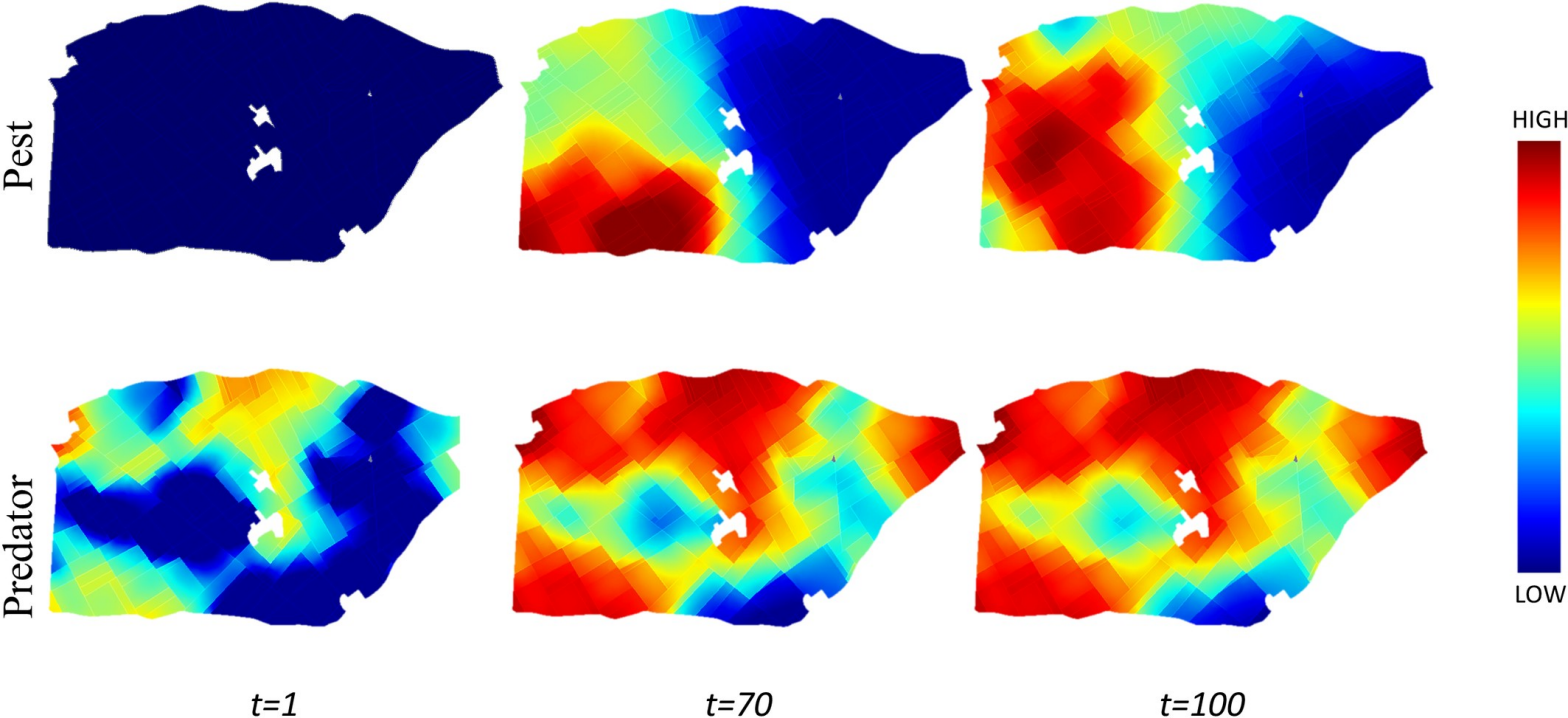
Snapshots of pest and predator spatial dynamics. Simulation of predator-pest population dynamics at different time intervals *t = {1*, *70*, *100}*. At the initial stage, the pest density (first line) is very low, followed by random introduction of pest. As time proceeds, the pest density increases (from left to right), and predator density (last line) also increases and diffuses to surrounding fields. At the final time step, high pest density arises where predators are absent.

We developed a spatially explicit predator-pest model based on a system of partial differential equations. The model is built on a previously developed model that considers both 2D diffusion on polygons and 1D diffusion on edges [11]. Simulations are performed over a [0,100]-time interval representing a cropping season with a time step of 1 day. The model parameters and their range of simulated values are reported in Table 2. Numerical simulations of the spatio-temporal partial differential equation system of predator-pest dynamics are performed using the Freefem++ finite-element framework [42]. The predator-pest dynamics is illustrated by snapshots at different time step (Fig 3) and by plots of the temporal dynamics in Figs B-D in S1 Text, and by S1 Video for the pest and S2 Video for the predator for the whole simulation period over the spatial domain.

#### 4.2.1 Predator dynamics

We model a generalist predator not showing strong aggregation behavior around pests. Hedges are the predator’s main habitat, which feeds on pests when moving into the fields. Using notations *t* for time and *x* for a spatial location, we thus assume the following 1-dimensional reaction-diffusion model for the predator density $v_{h_{i}}$ on the edge *h*_*i*_:

$$\left\{ \begin{array}{ll} \partial_{t}v_{h_{i}}=\partial_{xx}D_{1}^{v}v_{h_{i}}+r_{v}v_{h_{i}}\left(1-\frac{v_{h_{i}}}{K_{h_{i}}}\right) & \mathrm{if}\;\mathrm{the}\;\mathrm{edge}\;h_{i}\mathrm{carries}\;a\;\mathrm{hedge},\\ \;v_{h_{i}}=0 & \mathrm{otherwise}, \end{array}\right.$$ (2)

where $D_{1}^{v}$ is the diffusion parameter of the predator along hedges, *r*_*v*_ is the intrinsic growth rate of the predator, and $K_{h_{i}}$ is the carrying capacity of the hedge *i*. If two hedges are linked together at one of their endpoints, then the dynamics in Eq (7) apply continuously across the junction.

In addition, the predator forages on fields where it feeds on the pest. The population density $v_{\Omega_{i}}$ of predators in a field Ω_*i*_ is modelled by a reaction-diffusion equation with mobility parameter within field $D_{2}^{v}$, predating rate *β*, and life span 1/*m*_*v*_:

$$\partial_{t}v_{\Omega_{i}}=\Delta D_{2}^{v}v_{\Omega_{i}}-m_{v}v_{\Omega_{i}}+\beta u_{\Omega_{i}}v_{\Omega_{i}}$$ (3)

#### 4.2.2 Pest dynamics

We suppose that edges do not modify directly pest population dynamics. Writing $u_{h_{i}}$ for the pest density in an edge *h*_*i*_, we set

$$u_{h_{i}}=0\;for\;all\;i.$$ (4)

The pest is a specialist of the principal crop and, without dispersal, it shows positive growth only in crop fields. The bidimensional reaction-diffusion model for the pest density $u_{\Omega_{i}}$ in field $u_{\Omega_{i}}$ is

$$\left\{ \begin{array}{l} \;\partial_{t}u_{\Omega_{i}}=\Delta D_{2}^{u}u_{\Omega_{i}}+r_{u}u_{\Omega_{i}}\left(1-\frac{u_{\Omega_{i}}}{C_{it}}\right)-\beta u_{\Omega_{i}}v_{\Omega_{i}}\;\mathrm{for}\;\Omega_{i}\;\mathrm{with}\;\mathrm{crop},\\ \partial_{t}u_{\Omega_{i}}=\Delta D_{2}^{u}u_{\Omega_{i}}-m_{u}u_{\Omega_{i}}-\beta u_{\Omega_{i}}v_{\Omega_{i}}\;\mathrm{for}\;\Omega_{i}\;\mathrm{with}\;\mathrm{non}‐\mathrm{crop}, \end{array}\right.$$ (5)

where $D_{2}^{u}$ is the diffusion parameter of the pest in fields, *r*_*u*_ is its intrinsic growth rate on crop category, *β* is the predating rate, and 1/*m*_*u*_ is the life span of the pest on non-crop fields.

In a crop field, a pesticide application is performed when the average pest population density in that field exceeds a given threshold, which we here fix to 0.2 pests km^-2^. Pesticide applications strongly reduce the carrying capacity *C*_*it*_ of the field *i* (Eq (5)):

$$\left\{ \begin{array}{l} C_{it}=K_{\Omega_{i}}\;\mathrm{if}\;\mathrm{no}\;\mathrm{pesticide}\;\mathrm{application}\;\mathrm{is}\;\mathrm{applied},\\ C_{it}=\frac{K_{\Omega_{i}}}{200}\;\mathrm{during}\;\mathrm{the}\;\mathrm{period}\;e_{t}\;\mathrm{for}\;\mathrm{which}\;\mathrm{the}\;\mathrm{pesticide}\;\mathrm{application}\;\mathrm{is}\;\mathrm{efficient}. \end{array}\right.$$ (6)

This results in a pesticide application efficacy providing a 99.5% pest reduction, which can be considered an ideal-optimal case in practice. More realistic values of pesticide application efficacy should be around 70% [43,44]; this alternative scenario is analyzed in S1 Text, where the sensitivity to the pesticide application threshold is also tested.

Instead of a applying a reduction of the carrying capacity, we could have used an additional linear mortality term to account for the effects of pesticide applications, but this would have implied the modification of both growth and carrying capacity. For that reason, and to keep the model parsimonious, possible effects of pesticide applications are assumed to change only the carrying capacity (Eq 6), which is equivalent to using quadratic additional mortality term.

We point out that here we set the carrying capacity as a general saturation level for pest and predator densities, but it does not necessarily correspond to the number of individuals per *km*^2^. Similarly, mortality other than for predating or pesticide applications could have occurred in crop fields, but we have opted against this option for the sake of parsimony.

#### 4.2.3 Coupling predator-pest dynamics over the entire landscape

Using the framework described in [11], the dynamics described by Eqs (2) to (6) are coupled over the full landscape using the following assumptions (see S1 Text): (i) edges (with or without a hedge) do not represent a barrier for the pest, (ii) edges without a hedge do not represent a barrier for the predator, (iii) the predator is attracted by hedges, thus migration from fields to hedges (*ρ*_21_) is relatively high, (iv) the predator shows aversion to move outside its natural habitat, thus migration from hedges to fields (*ρ*_12_) is lower than migration from fields to hedges. We consider reflecting conditions on landscape boundaries, meaning that in- and out-fluxes between the landscape and its surrounding environment are equal.

Since pest population grows in crop habitat but not in non-crop habitat in our model, an increase in pest density with a higher crop proportion is expected. Similarly, since predators prefer hedges, higher hedge proportion favours predator movement through the landscape, thus, increasing predator density and predating pressure.

### 4.3 Pest arrival and spatio-temporal design

Initially, the predator is present in all hedges at carrying capacity. The pest is introduced randomly in space and time. The average number of pest inoculations in a single simulation is proportional to the proportion of crop field area in the landscape, and we draw the actual number of inoculations from a Poisson distribution. The maximal average number of pest inoculations is 25 and arises when the crop is grown in all fields. Inoculated crop fields are picked at random with probability depending on their relative surface.

### 4.4 Statistical methoh2ds for analyzing simulation outputs

We define an experimental design based on Sobol’s sequences leading to 11,500 distinct parameter configurations [45–47]. For each parameter combination, we consider 15 landscape replicates, leading to a total of 172,500 simulations. We first conduct a Sobol sensitivity analysis on the mean and standard deviation of predator density, pest density and number of pesticide applications by averaging the outputs over landscape replicates and crop fields. First-order indices were estimated with Sobol–Saltelli’s method [48,49], whereas total indices are estimated with Sobol–Jansen’s method [48,50]. These analysis are performed within the R software version 3.0.3 (R Team, 2003), using the packages fOptions (v. 3010.83) and sensitivity (v. 1.11).

Then, to further explore direction and magnitude of variations in response variables with respect to landscape parameters, we applied Generalized Linear Models (GLMs). Pest and predator densities, and pesticide application numbers (if different from 0), are analysed as response variable by using the Gamma distribution with log-link function. Additionally, presence/absence of pesticide applications during a simulation is analyzed using a GLM with binomial distribution. We develop GLM formulas containing covariable interactions (see Table 2) up to second order, and we use a step-wise variable selection algorithm based on the Bayesian Information Criterion (BIC) in order to iteratively select the “best subset” of variables for each model.

Finally, we use Generalized Linear Mixed-Effect models to analyze occurrences of pesticide applications by taking into account their spatial position in the landscape. We use the log-transformed area (*Area*) and perimeter (*Perimeter*) to take into account the geometrical properties of the fields, and we use the number of adjacent crop fields (*Adj*_*C*_), the number of adjacent hedges (*Adj*_*H*_), and the number of pesticide applications applied in the adjacent crop fields (*Adj*_*Tr*_) to take into account the composition and dynamics in local neighbourhoods. In addition, we include the estimated linear effects from the global models as offsets. The random effect is structured by the landscape simulation to account for its specific dynamics. By analogy with the global GLMs, the presence/absence of pesticide applications is analyzed using the binomial response distribution, and numbers of pesticide applications are analyzed with the Gamma distribution for the response variable with a log-link function. Again, we consider predictor interactions up to 2^nd^ order. These analyses are performed using the R package lme4 with R version 3.2.3 [51].

## Supporting information

S1 Text Supplementary Information of the paper: “More pests but less pesticide applications: ambivalent effect of landscape complexity on conservation biological control”. More details about the model and results.
(PDF)

S1 Video Supplementary video of pest dynamic: a video illustrating an example of spatio-temporal pest dynamics. (GIF)

S2 Video Supplementary video of predator dynamic: a video illustrating an example of spatio-temporal predator dynamics. (GIF)
