# Supplementary material for: More pests but less pesticide applications: Ambivalent effect of landscape complexity on conservation biological control
Source: PLoS Comput Biol. 2021 Nov 8;17(11):e1009559. doi: 10.1371/journal.pcbi.1009559 (PMC8601610; doi:10.1371/journal.pcbi.1009559)
Supplement: S1 Text — More details about the model and results. (PDF) [file pcbi.1009559.s001.pdf]

S1 Supplementary Information of the paper: "More pests but less pesticide applications: ambivalent effect of landscape complexity on conservation biological control"

1. Description of the 2D/1D model for population dynamics in the landscape

Here, we detail the description of the dynamics of a species in a landscape defined as a 2D matrix crossed by 1D corridors, following the methodology developed in [1]. Here, we report some of the key information to understand the 2D/1D model for population dynamics for our analysis. More details can be found in the original paper [1].

2D reaction-diffusion equations describe the dynamics in the matrix, and another set of 1D reaction-diffusion equations describe the dynamics in the corridors. The fluxes among the matrix and the corridors are described by coupling terms between the two sets of equations.

We consider a 2D matrix defined by a set $\Omega \subset R^2$, composed of finite mosaics i of polygonal disjoint 2D patches Ω_i separated by corridors (S1A Fig). Patch boundary is denoted by $\delta\Omega_i$, each boundary consisting of a finite number of 1D edges λ_i^k . The edges can be classified as: interior edges (= the corridors), and exterior edges which belong to the boundary $\delta\Omega$ of Ω , for which no particular 1D dynamics are modelled. The population density is denoted by v_i in each patch Ω_i and by u_i^k in each corridor λ_i^k.

.

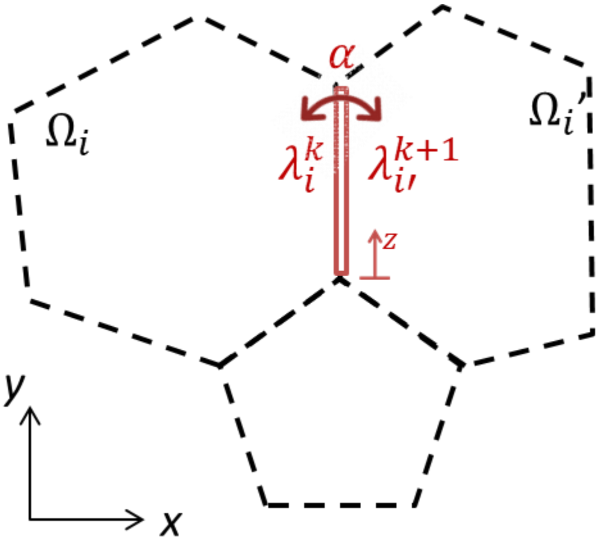

S1A Fig. Landscape representation. Patches $\boldsymbol{\Omega_i}$and edges $\boldsymbol{\lambda_i^k}$ at patch boundaries.

1.1 Dynamics in the 2D matrix

The population density is modelled by a reaction-diffusion equation:

$$\delta_t v_i = d\Delta v_i + f(v_i),$$

where d is the diffusion parameter that describes the mobility in the matrix 2D, and f is the growth function that describes the birth and death events in the patch Ω_i.

The exchanges among patch Ω_i and the surrounding corridors are described by the flux terms:

$$d\nabla v_i\, \boldsymbol{n} = \rho_{12} u_i^k(t,x,y) - \rho_{21} v_i\ (t,x,y),$$

where $\rho_{12} u_i^k(t,x,y)$ describes the flux of individuals leaving the corridor $\boldsymbol{\lambda_i^k}$ and entering the patch $\boldsymbol{\Omega_i}$ at time t and at the position (x,y), and $\rho_{21} v_i\ (t,x,y)$ describes the flux of individuals leaving the patch $\boldsymbol{\Omega_i}$ and entering the corridor $\boldsymbol{\lambda_i^k}$; finally, $\boldsymbol{n} = \boldsymbol{n}(\boldsymbol{x},\boldsymbol{y})$ denotes the outward unit normal to the boundary $\delta\Omega_i$. On the exterior boundary edges $\boldsymbol{\lambda_i^k} \in \delta\Omega_i$, standard reflecting boundary conditions are assumed: $d\nabla v_i\, \boldsymbol{n} = 0$. These boundary conditions mean that the individuals hitting the boundaries are reflected back inside the domain.

1.2 Dynamics in the corridors

Each corridor $\boldsymbol{\lambda_i^k}$ belongs to the common boundary of Ω_i and of another set, which is denoted by $\Omega_{i'}$, *i.e.*, $\boldsymbol{\lambda_i^k} = \boldsymbol{\lambda_{i'}^{k\prime}}$, where we model the 1D dynamics on each side of the corridor. The population densities in the corridor can be denoted by u_i^k and $u_{i'}^{k\prime}$ from the Ω_i and the $\Omega_{i,}$ sides, respectively, and we assumed that $u_i^k \neq u_{i'}^{k\prime}$, in general. The exchanges between the two sides of the corridor are taken into account through a permeability parameter α > 0 (S1A Fig). To state the 1D equation for the dynamics in the corridors, we define an isometric transformation $z \to \big(x(z), y(z)\big)$, which maps any corridor $\boldsymbol{\lambda}$ into an interval (0, $L(\boldsymbol{\lambda})$), where $L(\boldsymbol{\lambda})$ is the length of the corridor. Thus, the population density in the new coordinate $z \in L(\lambda)$ is defined by $\tilde{u}(t,z) = u(t,x,y)$. The population dynamics in each corridor $\boldsymbol{\lambda_i^k} = \boldsymbol{\lambda_{i'}^{k\prime}}$ separating two patches Ω_i and $\Omega_{i'}$ are described as follows:

$$\delta_t \tilde{u}_i^k = D\delta_{zz}\tilde{u}_i^k + \rho_{21} v_i\ \big(t, x(z), y(z)\big) - \rho_{12}\tilde{u}_i^k(t,z) - \alpha\tilde{u}_i^k(t,z) + \alpha\tilde{u}_{i'}^{k'}(t,z) + g\big(\tilde{u}_i^k\big),$$

$$t > 0, \qquad z \in \Big(0, L\big(\boldsymbol{\lambda_i^k}\big)\Big),$$

where g is the growth function in the corridor $\boldsymbol{\lambda_i^k}$; α is permeability parameter among the two side of the corridors, and D is the diffusion parameter on 1D corridor.

2. Predator and Pest dynamic

Here we show an example of the pest-predator dynamics resulting from the landscape configuration showed in S1B Fig. S1C Fig illustrates snapshots for different time steps showing the pest and predator density in the whole landscape. S1D Fig shows temporal dynamics of pest and predator aggregated in space. In addition, we provide two video files of .gif type to show the whole spatio-temporal dynamics from the same simulation as in S1B-D Figs (See S1 and S2 Video).

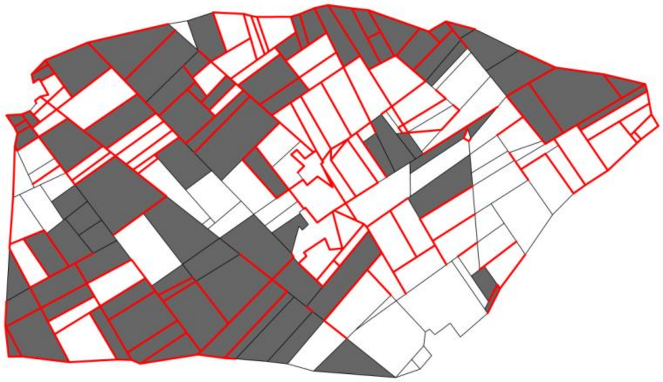

S1B Fig. Spatial configuration of crop (in grey) and hedges (in red).

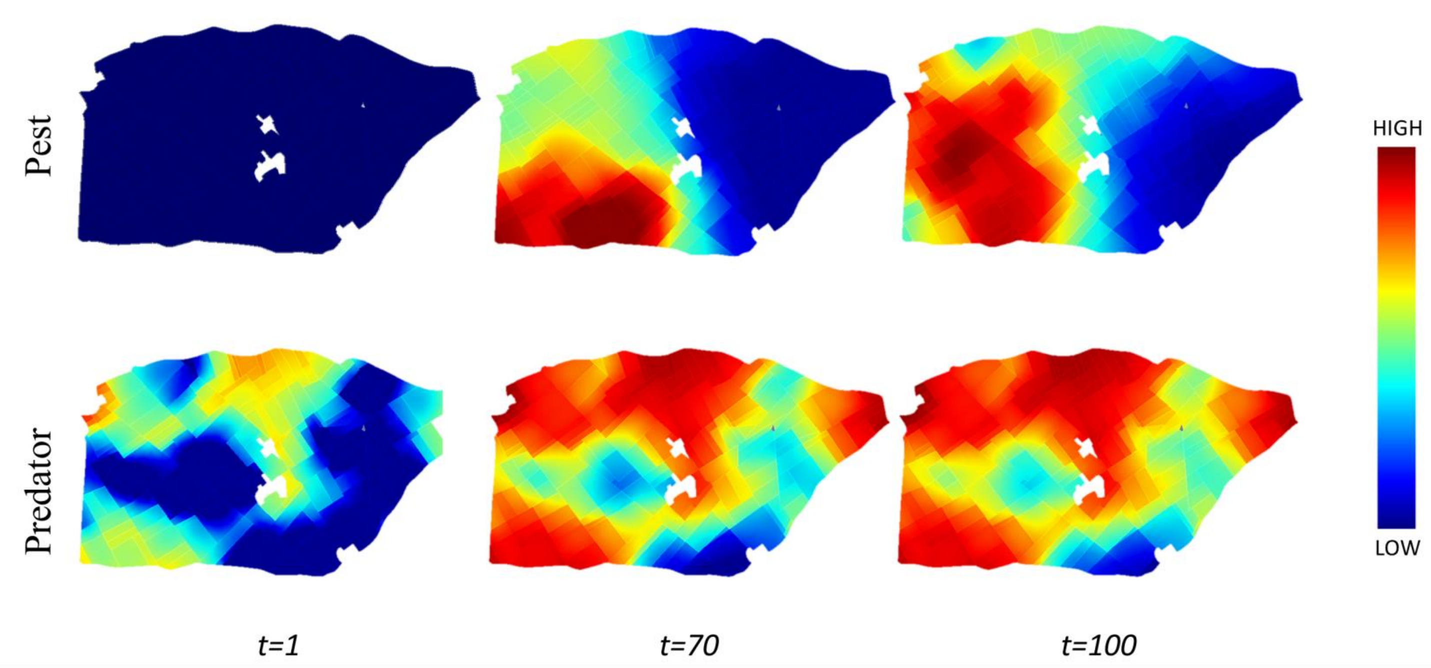

S1C Fig. Snapshots of pest and predator spatial dynamics. Simulation of predator-pest population dynamics at different time intervals <u>*t={1, 70, 100}.*</u> At the initial stage, the pest density (first line) is very low, followed by random introduction of pest. As time proceeds, the pest density increases (from left to right), and predator density (last line) also increases and diffuses to surrounding fields. At the final time step, high pest density arises where predators are absent. The temporal dynamic is shown in S1D Fig.

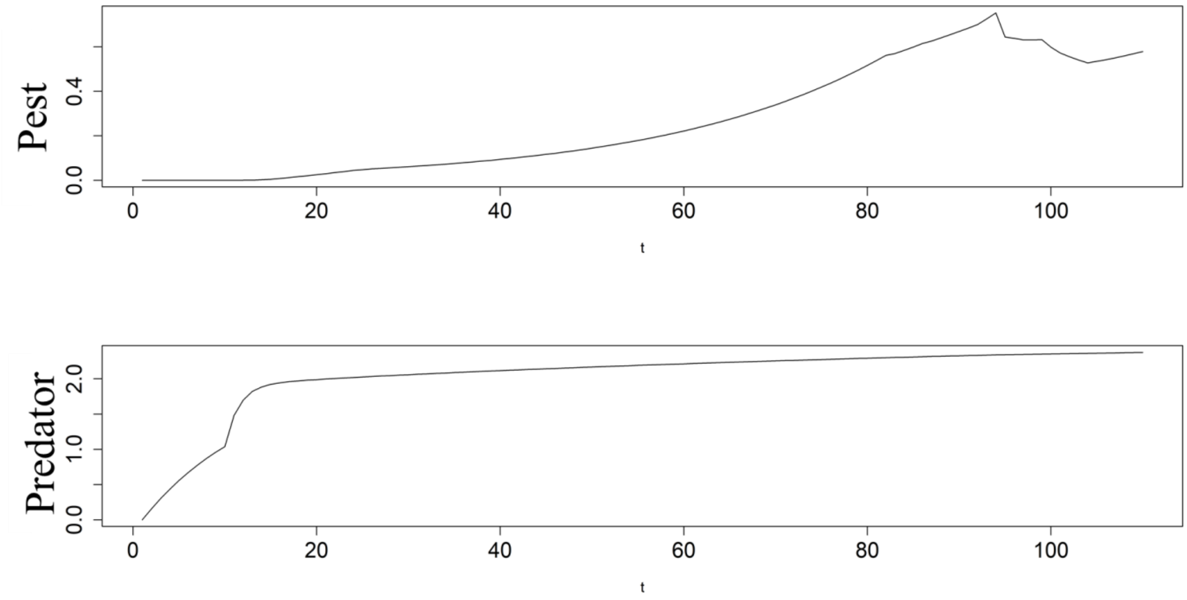

S1D Fig. Temporal dynamic of pest (first line) and predator (second line), aggregated over space.

3. Complete Sobol sensitivity analysis for predator and pest density and pesticide applications

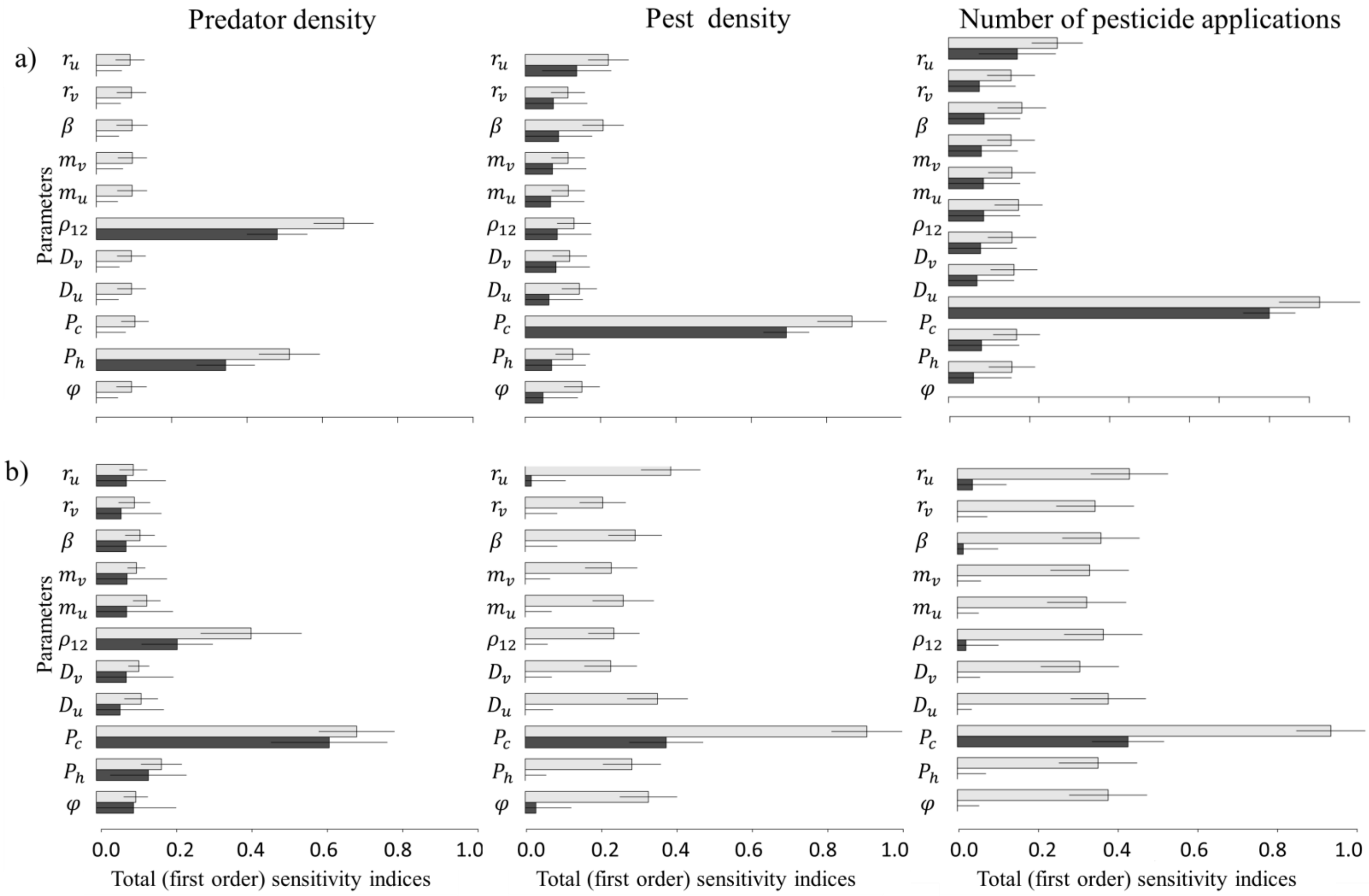

S1E Fig. Sobol sensitivity analysis. Total sensitivity indices (grey bar) and first-order sensitivity indices (black bar) of space-time averaged values for predator density, pest density and number of pesticide applications based on the mean (Panel a) or on the variance (Panel b) calculated over replicated simulations. The length of the bar indicates the mean of the sensitivity index, and the solid line indicates its 95% confidence interval.

3. Estimated effects of Generalized Linear Models (GLMs) for pest and predator densities, and for presence/absence and number of pesticide applications, and Generalized Linear Mixed-Effect Model for presence/absence and number of local pesticide applications.

a)

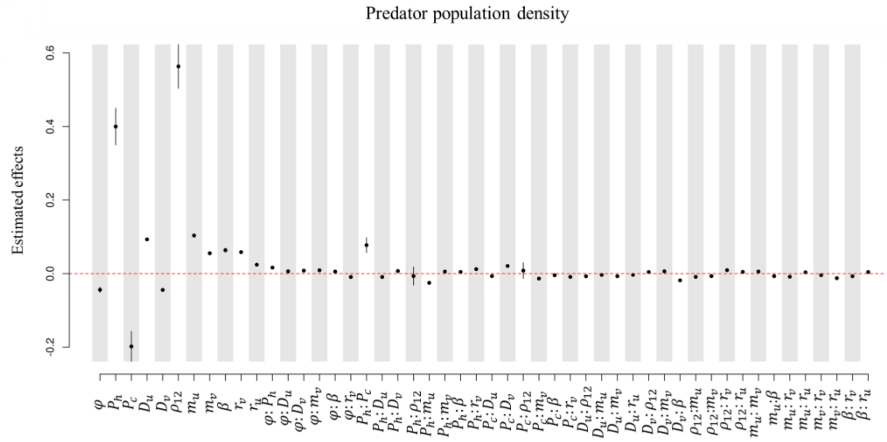
Predator population density
Estimated effects

b)

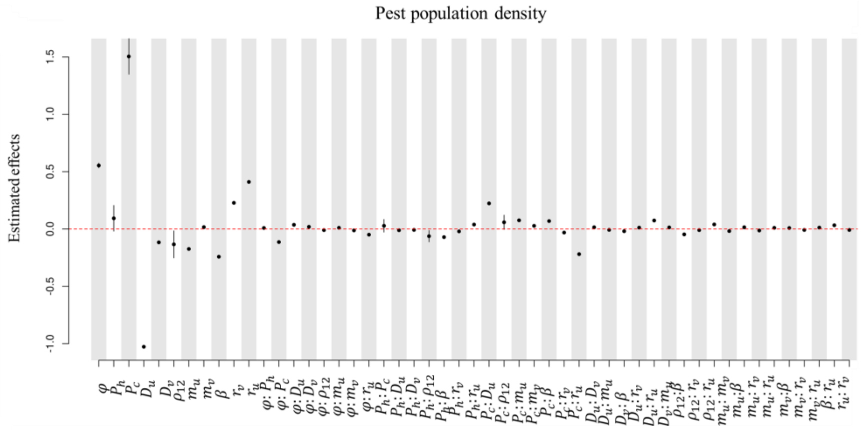
Pest population density
Estimated effects

c)

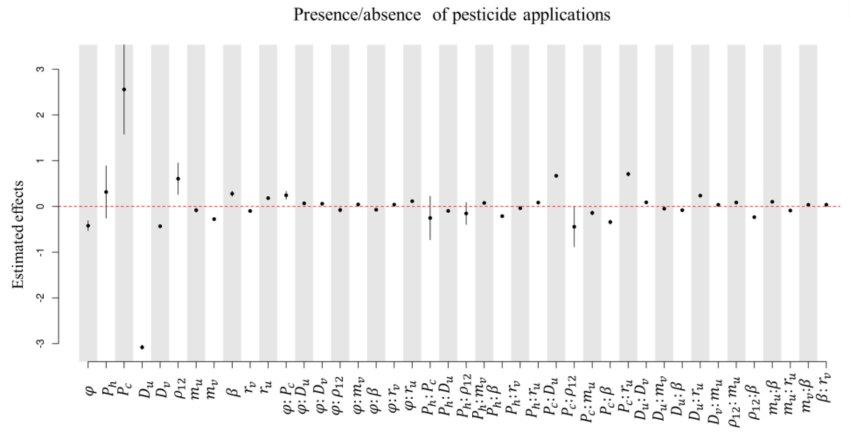
Presence/absence of pesticide applications
Estimated effects

d)

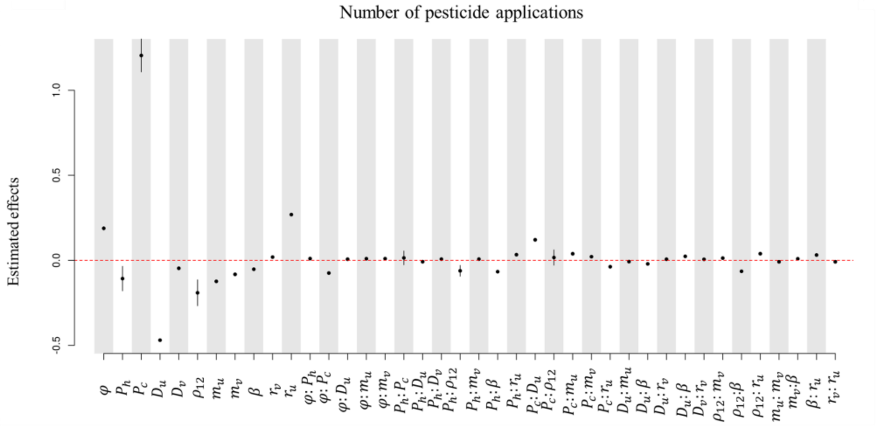
Number of pesticide applications
Estimated effects

S1F Fig. GLM coefficient estimates. Effects of input parameters and their bivariate interactions on pest and predator population dynamics: Coefficient estimates (dots) and their confidence intervals (segments) for the parameters retained by the stepwise selection in the GLM for the predator density (a), the pest density (b), the presence/absence of pesticide applications (c) and the number of pesticide applications (d).

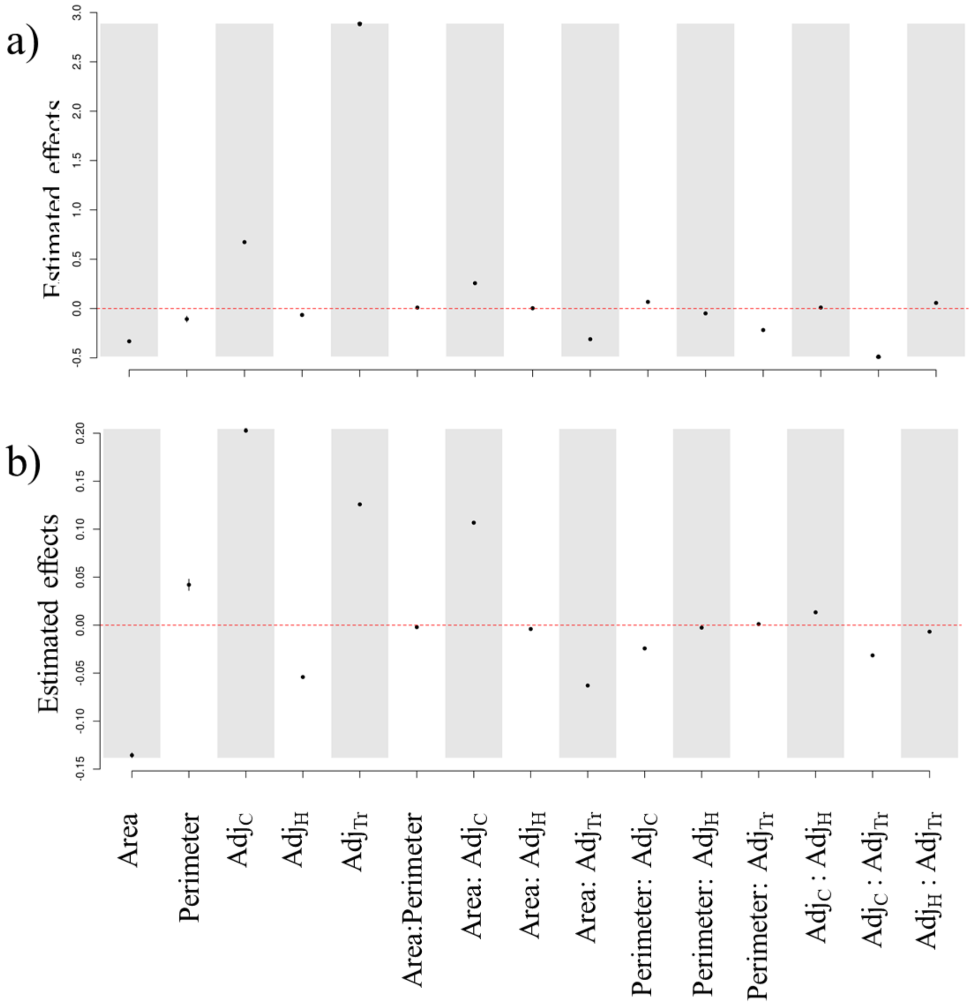

S1G Fig. Generalized Linear Mixed-Effect coefficient estimates. Estimated local effects (dots) and confidence intervals (segments) for the presence/absence of pesticide applications (a) and for the number of pesticide applications (b). The intercept values are not shown in this plot to better focus on the effects of the landscape covariables.

4. Sensitivity to pesticide application parameters

We test the sensitivity of our findings to pesticide applications when varying the pesticide efficacy and application threshold. We consider the following contrasted scenarios at landscape level:

- Scenario S1: a baseline scenario that considers an optimal pesticide efficacy (reduction of 99.5% of pest, C_{it} = 0.1 pests km^{-2} after application (See Table 2)) and a low application threshold (0.2 pests km^{-2});
- Scenario S2: sub-optimal pesticide efficacy (reduction of 70% of pest, C_{it} = 6 pests km^{-2} after application) and a low application threshold (0.2 pests km^{-2});
- Scenario S3: optimal pesticide efficacy (reduction of 99.5% of pest, C_{it} = 0.1 pests km^{-2} after application (See Table 2)) and a low application threshold (2 pests km^{-2}).

Scenario S1 depicts an ideal context for pesticide efficiency, while more realistic pesticide applications inflict about 70% mortality to pests [2,3]. The pesticide application threshold controls the pesticide application frequency, which could be highly variable depending on the pest species and on the economic value of crops [4]. Thus, a very low threshold generates a high pesticide application frequency, while a high threshold leads to more moderate pesticide utilisation.

We defined a smaller experimental setting where we vary only landscape parameters (aggregation (ϕ), crop proportion (Pc), hedge proportion (P_h)), and we perform a complete plan of 4 levels using the same ranges presented in Table 2 of the main text, with 15 repetitions each. We fixed population parameters (Table 2 in the main text) to the median of the values considered in the main paper. For each scenario, we obtain 960 simulations.

To contrast the effects of these different scenarios on CBC, we applied GLMs considering the scenarios along with the landscape variables. Pest and predator densities, and pesticide application numbers (if different from 0), are analyzed as response variable by using the Gamma distribution with log-link function; presence/absence of pesticide applications during a simulation is analyzed using a GLM with binomial distribution. We use the same GLM formulas as those presented in the main text containing covariable interactions (see Table 2) up to 2nd order, and we also use a step-wise variable selection algorithm based on the Bayesian Information Criterion (BIC) in order to select the "best subset" of variables for each model.

Results are presented in S1H Fig. In general, we observe that the directions of estimated effects are maintained across the scenarios, while the mean estimated effect has magnitude depending on the scenario. As expected, scenario S2 leads to an increase of pesticide applications, where covariates favoring pest outbreaks show a stronger effect than S1 (*e.g*, crop proportion for pest density (S1Hb Fig) and presence/absence of pesticide applications (S1Hc Fig)). Scenario S3 is expected to reduce the pesticide application frequency; therefore, for covariates favoring pest outbreaks, we observe that the estimated effect value is lower than S1 (*e.g,* spatial aggregation (ϕ) and crop proportion (Pc), for presence/absence of pesticide applications (S1Hc Fig)). Interestingly, S2 and S3 show remarkable effects provided by hedge proportion (Ph) for pest density: in S1, hedge proportion (Ph) is not significant, while in S2 and S3 hedge proportion (Ph) has a significant negative effect. Indeed, when pest reduction is lower due to low pesticide efficacy, or when pest reduction is slower due to an elevated pesticide application threshold on pest density, hedges may show a more relevant role in slowing down pest dynamics.

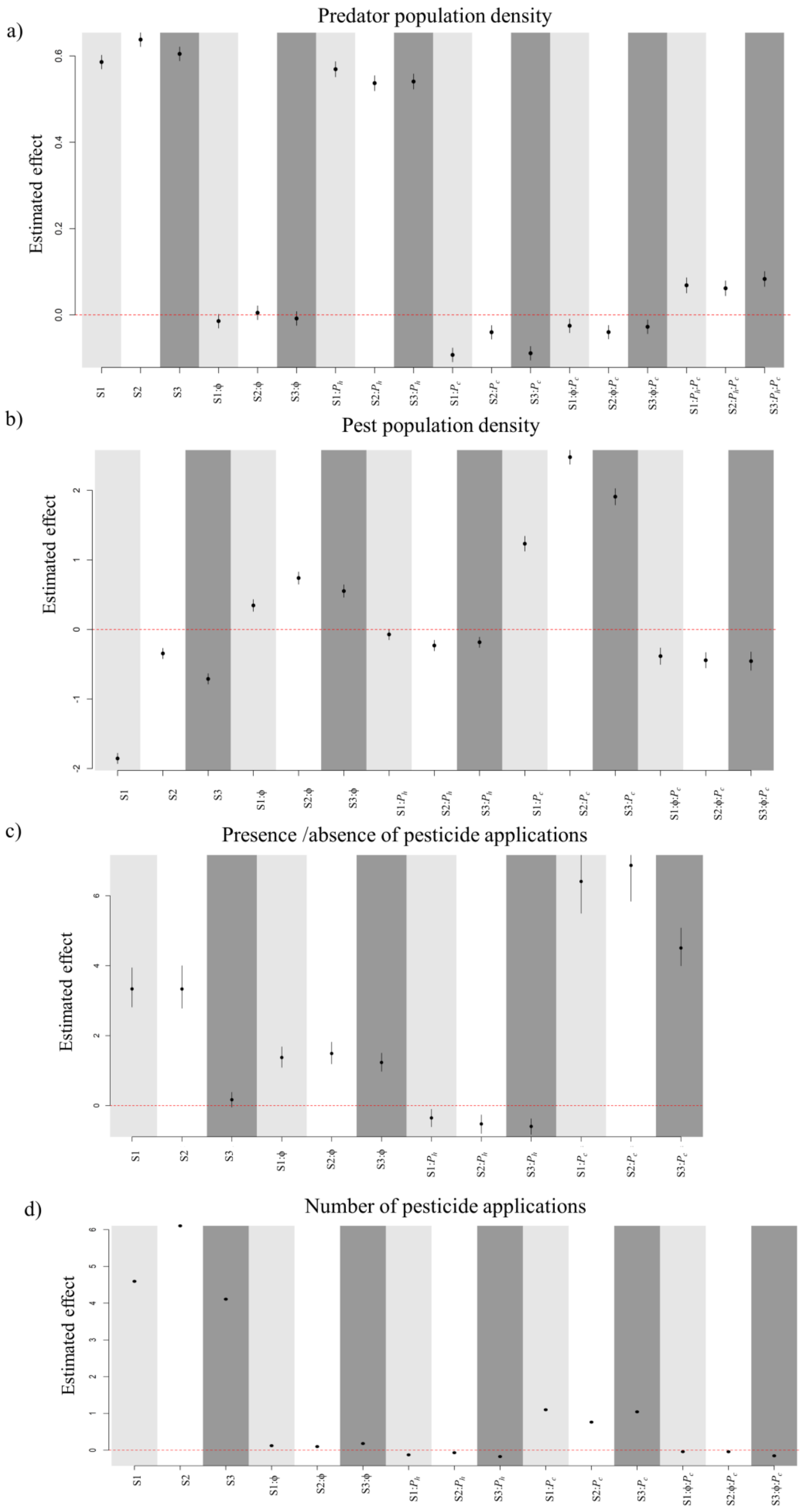
a)
Predator population density
Estimated effect
b)
Pest population density
Estimated effect
c)
Presence /absence of pesticide applications
Estimated effect
d)
Number of pesticide applications
Estimated effect

S1H Fig. GLM coefficient estimates for each scenario in the analysis of pesticide application sensitivity. Effects of input parameters and their bivariate interactions on pest and predator population dynamics: Coefficient estimates (dots) and their confidence intervals (segments) for the landscape parameters retained by the stepwise selection in the GLM, for the predator density (a), the pest density (b), the presence/absence of pesticide applications (c), and the number of pesticide applications (d). Scenarios S1, S2, S3 are indicated through different gray scales. All values are significant except for the following: *S1:* ϕ, *S2:* ϕ, *S3:* ϕ in a); *S1:*P_h in b).

Bibliography

1. Roques L, Bonnefon O. Modelling Population Dynamics in Realistic Landscapes with Linear Elements: A Mechanistic-Statistical Reaction-Diffusion Approach. PLoS One. 2016;11(3):e0151217.
2. Neil KA, Gaui SO, Mcrae KB. Control of the English grain aphid [Sitobion avenae (F.)] (Homoptera: Aphididae) and the oat-birdcherry aphid [Rhopalosiphum padi (L.)] (Homoptera: Aphididae) on winter cereals. Can Entomol. 1997;129(6):1079–91.
3. Abo El-Ghar GES, Abd AE. Impact of two synthetic pyrethroids and methomyl on management of the cabbage aphid, brevicoryne brassicae (L.) and its associated parasitoid, diaeretiella rapae (M'Intosh). Pestic Sci. 1989;25(1):35–41.
4. Bianchi FA, Ives AR, Schellhorn NA. Interactions between conventional and organic farming for biocontrol services across the landscape. Ecological Applications. 2013 Oct;23(7):1531-43.
