# Supplementary figures and images for: More pests but less pesticide applications: Ambivalent effect of landscape complexity on conservation biological control
Source: PLoS Comput Biol. 2021 Nov 8;17(11):e1009559. doi: 10.1371/journal.pcbi.1009559 (PMC8601610; doi:10.1371/journal.pcbi.1009559)

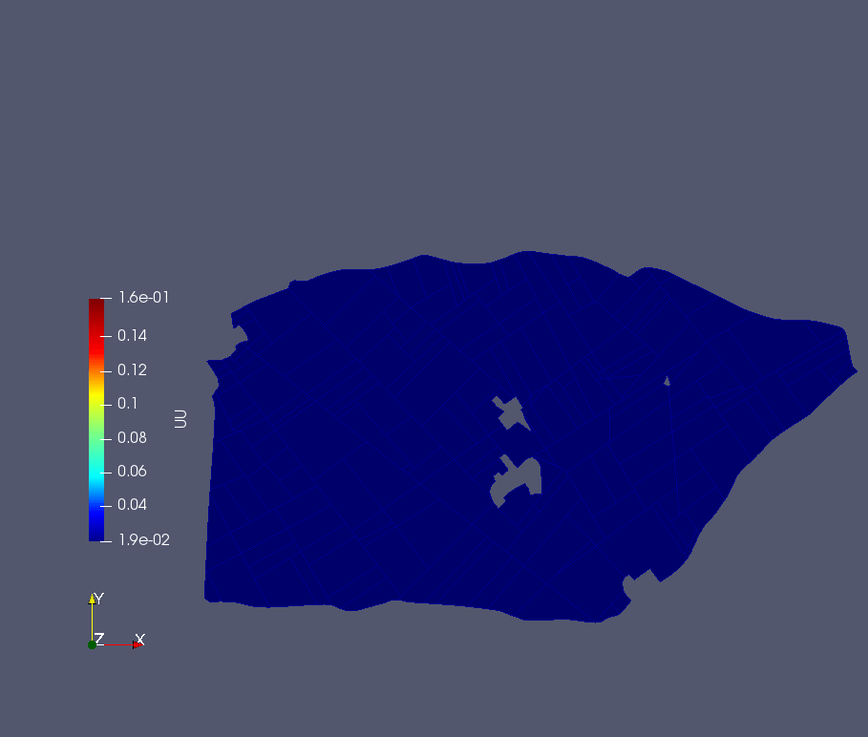

Supplement: S1 Video — (GIF) [file pcbi.1009559.s002.gif]

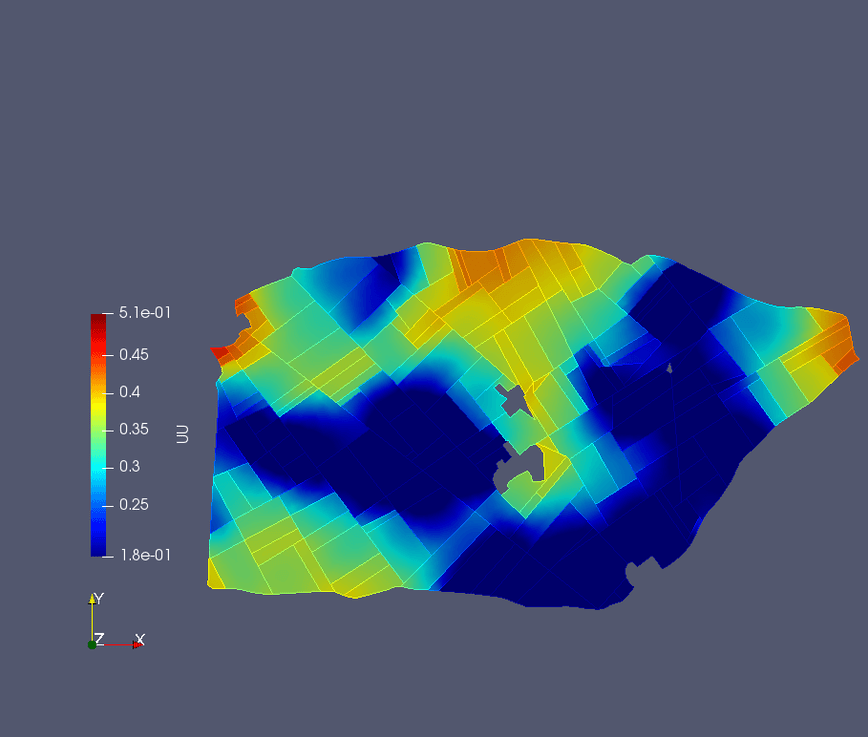

Supplement: S2 Video — (GIF) [file pcbi.1009559.s003.gif]
